# INSPIRE datahub: a pan-African integrated suite of services for harmonising longitudinal population health data using OHDSI tools

**DOI:** 10.3389/fdgth.2024.1329630

**Published:** 2024-01-29

**Authors:** Tathagata Bhattacharjee, Sylvia Kiwuwa-Muyingo, Chifundo Kanjala, Molulaqhooa L. Maoyi, David Amadi, Michael Ochola, Damazo Kadengye, Arofan Gregory, Agnes Kiragga, Amelia Taylor, Jay Greenfield, Emma Slaymaker, Jim Todd, INSPIRE Network

**Affiliations:** ^1^ Department of Population Health, Faculty of Epidemiology and Population Health, London School of Hygiene and Tropical Medicine, University of London, London, United Kingdom; ^2^ African Population and Health Research Center (APHRC), Nairobi, Kenya; ^3^ UNICEF (Malawi), Lilongwe, Malawi; ^4^ South African Population Research Infrastructure Network (SAPRIN), South African Medical Research Council, Durban, South Africa; ^5^ Department of Economics and Statistics, Kabale University, Kabale, Uganda; ^6^ Committee on Data of the International Science Council (CODATA), Paris, France; ^7^ Malawi University of Business and Applied Sciences, Blantyre, Malawi; ^8^ Implementation Network for Sharing Population Information from Research Entities (INSPIRE Network), Nairobi, Kenya

**Keywords:** data hub, data harmonisation, Common Data Model (CDM), OMOP CDM, longitudinal population health data

## Abstract

**Introduction:**

Population health data integration remains a critical challenge in low- and middle-income countries (LMIC), hindering the generation of actionable insights to inform policy and decision-making. This paper proposes a pan-African, Findable, Accessible, Interoperable, and Reusable (FAIR) research architecture and infrastructure named the INSPIRE datahub. This cloud-based Platform-as-a-Service (PaaS) and on-premises setup aims to enhance the discovery, integration, and analysis of clinical, population-based surveys, and other health data sources.

**Methods:**

The INSPIRE datahub, part of the Implementation Network for Sharing Population Information from Research Entities (INSPIRE), employs the Observational Health Data Sciences and Informatics (OHDSI) open-source stack of tools and the Observational Medical Outcomes Partnership (OMOP) Common Data Model (CDM) to harmonise data from African longitudinal population studies. Operating on Microsoft Azure and Amazon Web Services cloud platforms, and on on-premises servers, the architecture offers adaptability and scalability for other cloud providers and technology infrastructure. The OHDSI-based tools enable a comprehensive suite of services for data pipeline development, profiling, mapping, extraction, transformation, loading, documentation, anonymization, and analysis.

**Results:**

The INSPIRE datahub's “On-ramp” services facilitate the integration of data and metadata from diverse sources into the OMOP CDM. The datahub supports the implementation of OMOP CDM across data producers, harmonizing source data semantically with standard vocabularies and structurally conforming to OMOP table structures. Leveraging OHDSI tools, the datahub performs quality assessment and analysis of the transformed data. It ensures FAIR data by establishing metadata flows, capturing provenance throughout the ETL processes, and providing accessible metadata for potential users. The ETL provenance is documented in a machine- and human-readable Implementation Guide (IG), enhancing transparency and usability.

**Conclusion:**

The pan-African INSPIRE datahub presents a scalable and systematic solution for integrating health data in LMICs. By adhering to FAIR principles and leveraging established standards like OMOP CDM, this architecture addresses the current gap in generating evidence to support policy and decision-making for improving the well-being of LMIC populations. The federated research network provisions allow data producers to maintain control over their data, fostering collaboration while respecting data privacy and security concerns. A use-case demonstrated the pipeline using OHDSI and other open-source tools.

## 1 Introduction

Numerous population health research data harmonisation projects have been documented in the literature (1–7). However, many of those initiatives face limitations in achieving true Findability, Accessibility, Interoperability, and Reusability (FAIR) nature of data (8). For instance, while the CLOSER project (1) stands out as one of the most comprehensively documented projects in terms of making data FAIR, it relies on commercial tools for metadata development, rendering it impractical for deployment in resource-constrained settings. Additionally, its data integration mechanism lacks scalability.

Conversely, the Maelstrom project (3) and the Harmonisation project (9) have developed software solutions for data harmonization and the dissemination of multi-study data (10). These projects offer a systematic approach to data integration and adhere to data documentation standards, fostering FAIR-friendly data. Despite these advancements, challenges in scalability persist, posing potential hindrances to broader implementation and impact.

Data scalability refers to the capacity of a system, application, or infrastructure to smoothly manage growing volumes of data while maintaining optimal performance, responsiveness, and stability. Successful attainment of data scalability requires meticulous system design, thoughtful architectural decisions, and adept software implementation. Contemporary technologies, including cloud computing, containerization, microservices, and distributed & federated databases, offer instrumental tools and frameworks. These innovations empower the distribution of workloads and the efficient management of resources, thereby facilitating seamless data scalability.

The Network for Analysing Longitudinal Population-based HIV/AIDS Data on Africa (ALPHA) (4), the International Network for Demographic Evaluation of Populations and Their Health (INDEPTH) (11), and the South African Population Research Infrastructure Network (SAPRIN) (12) have successfully harmonised health and demographic surveillance data from longitudinal population studies (LPS) in Africa. These collaborative networks have effectively demonstrated the feasibility of harmonizing and analysing data from African LPS, yielding crucial demographic and epidemiological insights across the region. However, it is important to note that, while these harmonised datasets represent a significant step forward, their producers acknowledge that challenges related to scalability and the production of Findable, Accessible, Interoperable, and Reusable (FAIR) data are still at an early stage of development (13).

Advances in the systematic integration of Electronic Health Record (EHR) databases for research purposes have been propelled by common data models (CDM), exemplified by the Observational Medical Outcome Partnership (OMOP—https://ohdsi.org/data-standardization/) (14–16). These CDMs play a pivotal role in standardizing vocabularies and data structures, thereby facilitating large-scale analyses. The inherent scalability of CDMs is instrumental in consolidating disparate data sources. However, when viewed through a Findable, Accessible, Interoperable, and Reusable (FAIR) lens, CDMs exhibit a limitation—they lack sufficient contextual metadata to render the data easily discoverable and reusable (17).

This paper introduces the Implementation Network for Sharing Population Information and Research Entities (INSPIRE) datahub as a comprehensive solution for data integration and harmonisation. Functioning as a pan-African, federated research ecosystem, the datahub is designed to adhere to the principles of Findability, Accessibility, Interoperability, and Reusability (FAIR). It tackles critical issues related to reproducibility, standardization, and scalability of data harmonisation and sharing systems, primarily in low-resource settings, leveraging existing technologies customized for optimal use.

In our approach, we extend the utility of the Observational Medical Outcome Partnership Common Data Model (OMOP CDM) to accommodate African Longitudinal Population Studies (LPS) data. We incorporate structured metadata throughout the data pipeline, employing an Implementation Guide (IG) to ensure consistency. The paper outlines the process of ingesting and profiling data, mapping source data semantics to standard vocabularies for transformations into the OMOP CDM database. It also demonstrates the utilization of OMOP-based tools for analytics and the creation of well-structured provenance metadata accompanying the transformed data for sharing purposes.

By amalgamating the insights gathered from the experiences of ALPHA, INDEPTH, and SAPRIN in the African region, the scalability and standardization offered by the OMOP CDM, and adherence to FAIR data standards; the INSPIRE datahub emerges as a promising and sustainable mechanism for harmonizing data and facilitating analytics in resource-constrained settings. The INSPIRE network has strategically built upon the data harmonization efforts of the ALPHA network within the African region. This collaborative approach allowed INSPIRE to gain valuable insights into the challenges associated with data accessibility, confidentiality, administration, and various other aspects within the field.

## 2 Implementation

### 2.1 Virtualisation

The INSPIRE datahub is founded on the principles of virtualisation, a transformative technology that facilitates the development of virtual rather than physical versions of diverse computing resources. These resources encompass operating systems, servers, storage devices, and networks. Through virtualisation, multiple instances of these resources can operate independently on the same physical hardware, providing a flexible and efficient utilisation of computing infrastructure.

The adoption of virtualisation technology was driven by the goal of optimizing resource utilization, allowing multiple virtual instances to efficiently operate on a single physical server. Recognizing the critical need for resource efficiency in Africa's resource-constrained setups, this initiative was undertaken. Additionally, the pursuit of enhanced flexibility and scalability, crucial for accommodating dynamic workloads and evolving computing requirements at Longitudinal Population Studies (LPS) sites in Africa, guided the implementation. The consideration of disaster recovery capabilities, allowing swift replication and restoration of virtualised environments, further underscored the significance of this technological shift. Furthermore, virtualisation strengthens security by effectively isolating virtual instances. In summary, the multifaceted advantages of virtualisation render it essential for small-scale computing environments, providing not only efficiency and cost-effectiveness but also the adaptability and resilient disaster recovery capabilities essential for resource-constrained LPS sites in Africa.

Platform-as-a-Service (PaaS) (18) is a cloud computing service model that provides a comprehensive platform allowing users to develop, deploy, and manage applications without the complexities of infrastructure management. In the PaaS paradigm, users have access to a set of tools, services, and frameworks hosted on the cloud, enabling streamlined application development and deployment processes. PaaS eliminates the need for organizations to invest in and manage the underlying hardware and software infrastructure, allowing them to focus more on coding, testing, and deploying applications. This model enhances collaboration among development teams, accelerates time-to-market, and promotes scalability, making it a pivotal enabler for innovation and efficiency in the ever-evolving landscape of cloud computing. This approach was considered well-suited for resource-constrained Longitudinal Population Studies (LPS) sites in Africa, and the gradual adoption of such infrastructure, whether on public or private clouds, is gaining popularity in the region.

The INSPIRE data hub is a “platform-as-a-service” (PaaS) (18) hosted on the cloud, addressing issues of scalability, and streamlining basic administrative functions, including data backups. The initial prototype employed a commercial cloud service provider, and an evaluation was undertaken to determine the most suitable provider for an African data resource. The process of establishing the cloud infrastructure has been meticulously documented and is publicly accessible on the CODATA website under the page titled “INSPIRE Data Hub: Technical Results of Phase 1.” Within this page, comprehensive details of the setup components, the procedural steps involved, and the outcomes are recorded under the heading “INSPIRE EA IT Infrastructure on the Cloud” (19).

### 2.2 INSPIRE datahub architecture

**Figure 1:**
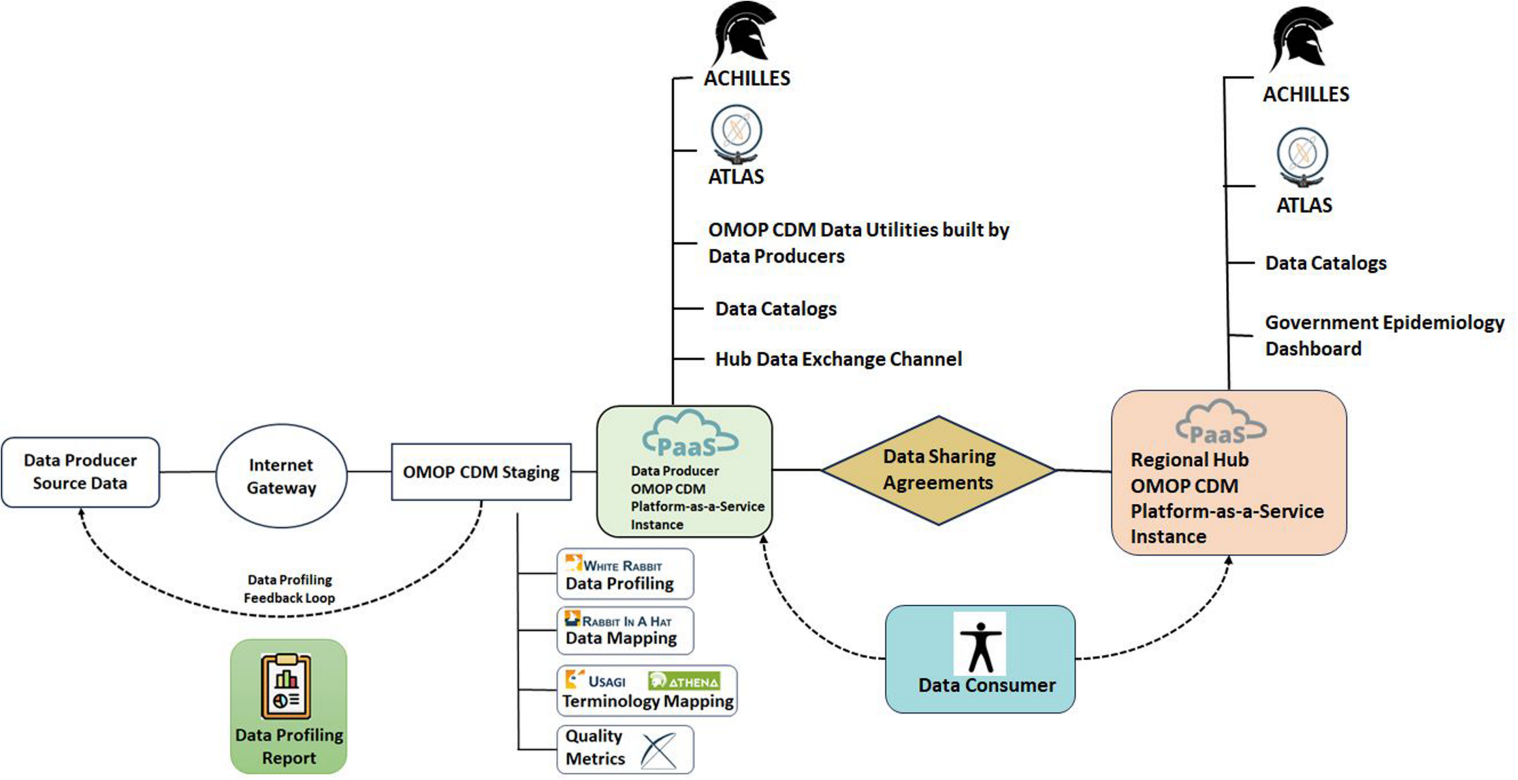
INSPIRE datahub overall architecture.

Figure 1 presents a detailed interpretation of the overall design of the INSPIRE datahub, showcasing the various components. This design, with its visual representation, serves as a key reference for understanding the structural and functional aspects of the datahub.

For a deeper exploration of this design, Greenfield has contributed an extensive explanation in a document titled “INSPIRE Data Hub: High-Level Architecture and Data and Metadata Flows” (20). This document provides a thorough breakdown of the high-level architecture, elucidating how data and metadata flow within the system. Greenfield's insights offer valuable context and insights into the intricate workings of the INSPIRE datahub.

Additionally, these details are part of the broader CODATA Decadal programme (21), ensuring that this information is not only accessible but also aligned with the long-term goals and strategies of CODATA. By referring to these resources, stakeholders, researchers, and interested parties can gain a comprehensive understanding of the datahub's design, its architectural intricacies, and the flows of data and metadata that emphasise its functionality.

### 2.3 OMOP CDM

**Figure 2:**
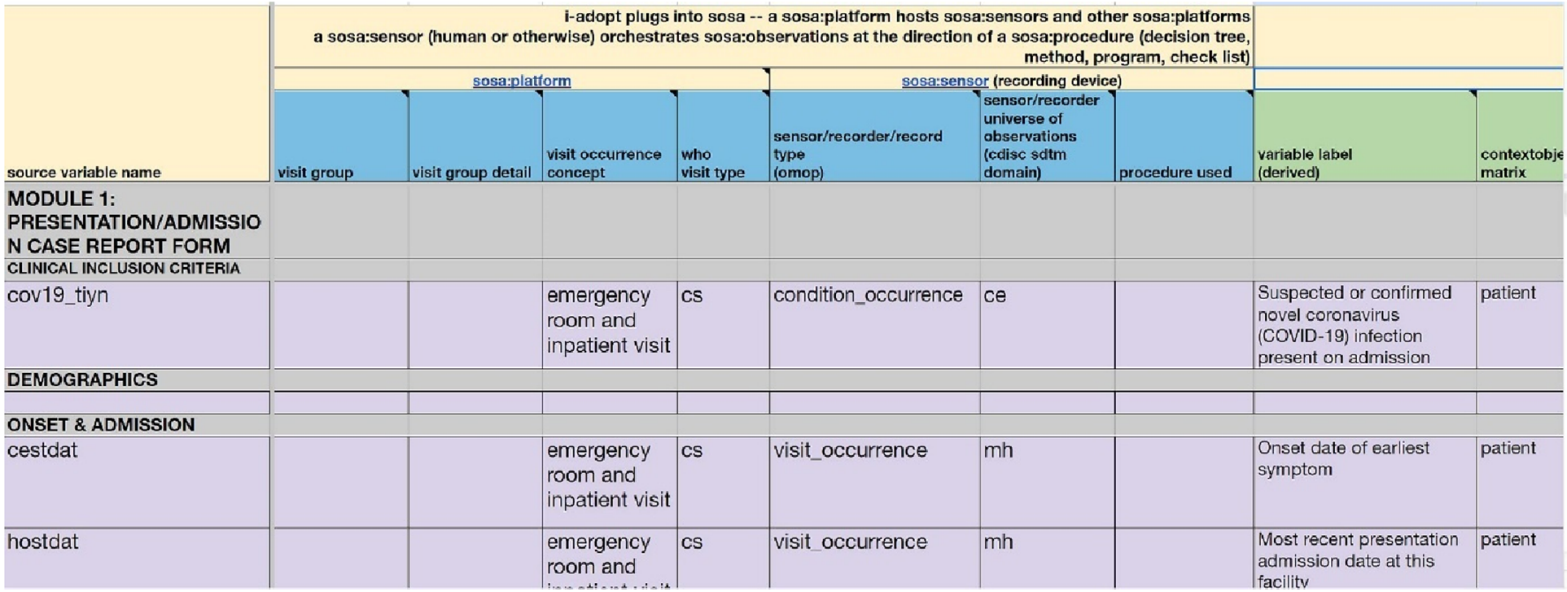
Demonstration metadata form for provenance documentation.

The Observational Medical Outcomes Partnership Common Data Model (OMOP CDM), described in the Book of OHDSI (22), stands as a framework in healthcare research, offering a standardised structure for organising and analysing health data from different sources. Developed to foster interoperability and consistency, OMOP CDM enables the integration of diverse healthcare data sources, such as electronic health records (EHR) and administrative claims, into a harmonised format. The model employs a set of standardised terminologies, ensuring semantic consistency and its relational database structure facilitates streamlined data analysis. OMOP CDM is useful for its ability to support large-scale observational studies, comparative effectiveness research, and other investigations requiring a comprehensive and standardised representation of healthcare data. Its adoption has significantly contributed to advancing evidence-based medicine and promoting collaborative research initiatives across diverse healthcare settings. A centralized repository for the standardised vocabularies and concepts is meticulously maintained in the Observational Health Data Sciences and Informatics (OHDSI) registry known as ATHENA, accessible at https://ATHENA.ohdsi.org/. The ATHENA registry plays a crucial role in ensuring consistency and coherence across diverse datasets by providing a standardised and centrally managed reference for vocabularies and concepts within the OHDSI framework. Figure 2 demonstrates a metadata form for the provenance documentation.

Initially considered for the integration of electronic health records (EHR), INSPIRE has adeptly extended the application of the Observational Medical Outcomes Partnership Common Data Model (OMOP CDM) to encompass African longitudinal population studies (LPS), leveraging data from Health and Demographic Surveillance Systems (HDSS) including demographic data and questionnaires (34). This versatile framework facilitates standard analyses through the utilisation of established OMOP-based tools. In addition, INSPIRE is augmenting the foundational CDM by introducing enhanced capabilities for provenance documentation, adhering to rigorous metadata standards. This proactive approach ensures a comprehensive and well-documented data stack, contributing to the robustness and transparency of analytical processes within the INSPIRE initiative.

### 2.4 Data mapping and migration

Interoperability, as defined by the Institute of Electrical and Electronics Engineers (IEEE), refers to the exchange of functional information between two or more systems for a specific purpose. Data mapping is a specific technique used to ensure interoperability by aligning data elements between two or more systems. It involves the identification and matching of data points in different systems, especially when the source and target systems use different terminologies or data structures. In health data, data mapping is an important process that involves establishing connections and equivalencies between data elements in different health information systems. It ensures that information from diverse sources, such as electronic health records, questionnaires (34), medical devices, health databases, or health surveillances can be integrated and exchanged. Data mapping is crucial for data harmonisation, by aligning data structures and transforming formats to achieve interoperability. This process allows researchers to effectively share, analyse, and utilise health information across various platforms.

Data mapping and migration encompass the following processes: (1) identification of the source and target data models, (2) identification of specific data elements (fields, attributes, columns) in both the source and target data models that require mapping, (3) determination of relationships between corresponding data elements in the source and target data models, (4) transformation of data to align with the target data model, including adjustments to codes, data types, formats, etc., (5) validation to ensure accurate representation of relationships in the transformed data with minimal or no data loss, and (6) documentation of the process for consistency and repeatability.

### 2.5 On-ramps

The process of bringing data into standardised databases involves many intricate procedures, ensuring a smooth integration of diverse data sources. This approach includes profiling, vocabulary mapping, variable and structure mapping, and the implementation of extraction, transformation, and loading (ETL) programs, combined with metadata ingestion services. Data and metadata management services play an important role in importing data into the platform, where it undergoes profiling using tools such as WHITERABBIT, which is an Observational Medical Outcomes Partnership (OMOP)-based tool (23).

After profiling, data mapping to OMOP tables is facilitated through dedicated tools such as RABBIT-IN-A-HAT (23), designed for OMOP-based data mapping. The task of terminology mapping is achieved using the OMOP tool USAGI (23), which is an offline tool for mapping. Also, mappings can be done online using the ATHENA vocabulary repository. This mapping can follow directly from the source data to OMOP or via community-defined data exchange protocols. Once mapping is complete, ETL processes come into play. An initial set of ETLs may be executed by data contributors, facilitating the migration of data from local systems into a standardised format through exchange protocols. Subsequently, a second set of ETLs may be applied, transitioning data from the exchange protocols to the OMOP format.

The platform accommodates various agreed-upon exchange protocols, supporting multiple data formats. In the realm of metadata, the INSPIRE team mandates minimal requirements, ensuring that a standardised set of metadata accompanies the submitted data, contributing to consistency and facilitating effective data management within the platform.

### 2.6 Off-ramps

The off-ramp services provide a gateway to hub data and metadata for use in a suite of tools from the Observational Health Data Sciences and Informatics (OHDSI) stack for data exploration, population characterisation, and advanced analytics. A standout tool within this repertoire is ACHILLES, offering robust capabilities for data exploration, quality assessments, and the generation of insightful summary statistics. Additionally, ATLAS can be included in this pipeline for low and no code data analysis.

The OHDSI stack features numerous other tools that harness the advantages of data structure and vocabulary standardisation, enabling scalable analytics. Furthermore, the platform allows for the development of personalized workbenches, empowering users to engage in tailor-made analytics to suit specific research needs.

INSPIRE acknowledges the importance of some types of research that operate based on static analysis datasets. These static datasets are important for purposes of disclosure risk control, citation, and reproducibility of findings. INSPIRE has developed a mechanism for meeting this need—the Immutable Cohort Data Store. The Immutable Cohort Data Store supports this requirement by providing an ability to recreate any given dataset drawn from the hub by persisting information about the state of the hub data when the cohort was applied. Such “immutable cohorts” can thus support the many functions that rely on the existence of static, unchanging versions of data sets. While not needed for all off-ramps (OHDSI applications provide their solutions to these problems) the ability to support different users’ needs requires this functionality.

### 2.7 Federated research network

Federated networks introduce a versatile paradigm wherein replicas of the Observational Medical Outcomes Partnership Common Data Model (OMOP CDM) platform can be instantiated at partner or collaborator sites. These federated databases mirror the structure of the parent OMOP CDM, offering access to standardised vocabularies and extraction, transformation, and loading (ETL) programs developed by the platform or the datahub. The deployment of common resources facilitates the on-ramping of individual-level data from sensitive sources into the federated database while maintaining stringent security measures to safeguard against external user access. Alternatively, partner or collaborative sites can perform their analyses and provide the results. In this regard, each OMOP CDM has a “results schema” that can be shared with the parent OMOP CDM to produce a combined set of results that spans the network.

The on-ramps serve as the gateway for describing metadata and exposing the cohorts available for analysis, with data owners having control over access permissions. Requests for analysis cohorts and/or their results can be made to the respective data owners. Once the analysis is conducted, the output data can be aggregated and seamlessly integrated into the broader analytical framework, aligning with similar data from diverse sources. This federated approach not only ensures the privacy and security of sensitive data but also enables collaborative and robust analyses by amalgamating insights from varied and distributed sources (24).

## 3 Use case: longitudinal, population-based residency data from the iSHARE collaboration in Africa

This study conducted a use case from the Health and Demographic Surveillance System (HDSS) to the Observational Medical Outcomes Partnership Common Data Model (OMOP CDM). The data was sourced from the INDEPTH Network Data Repository, accessible at https://indepth-ishare.org/index.php/catalog/central. The dataset curated by the members of the INDEPTH Network serves as a valuable repository of population and health data specifically tailored for Low- and Middle-Income Countries (LMICs). The observational data encompass demographic surveillance information, as detailed in Table 1. The specific datasets utilized in this analysis include the following: (1) Kenya—Nairobi HDSS INDEPTH Core Dataset 2003–2015 (Release 2018) (25), (2) Tanzania—Magu HDSS INDEPTH Core Dataset 1994–2012 Release 2015 (26), and (3) South Africa—Africa Health Research Institute INDEPTH Core Dataset 2000–2017 (Residents only)—Release 2019 (27).

**Table 1:** Description of variables in the INDEPTH core microdata.

Variable	Format	Description
RecNr	Numeric	A sequential number uniquely identifies each record in the data file
CountryId	Numeric	ISO 3166-1 numeric code of the country in which the surveillance site is situated
CentreId	Character	An identifier issued by INDEPTH to each member centre of the format CCCSS, where CCC is a sequential centre identifier and SS is a sequential identifier of the site within the centre in the case of multiple site centers
IndividualId	Numeric	A number uniquely identifying all the records belonging to a specific individual in the data file
Sex	Numeric	Sex of Individual
DoB	Character	The date of birth of the individual. Format: YYYY/MM/DD
EventCount	Numeric	The total number of events associated with this individual in this data set
EventNr	Numeric	A number increasing from 1 to EventCount for each event record in order of event occurrence
EventCode	Character	A code identifying the type of event that has occurred
EventDate	Character	The date on which the event occurred. Format: YYYY/MM/DD
ObservationDate	Character	The date on which the event was observed (recorded), is also known as the surveillance visit date. Format: YYYY/MM/DD
LocationId	Numeric	Unique identifier associated with a residential unit within the site and is the location where the individual was or became resident when the event occurred
MotherId	Numeric	The IndividualId of the mother. Only provided for BTH events
DeliveryId	Numeric	The RecNr of the delivery event associated with this birth

The INDEPTH Network conducted a comprehensive list of advanced analyses to delineate the characteristics of populations across its member sites. The dataset facilitated standard analyses, predominantly centered around event history. Key indicators, such as fertility rates, encompassed metrics like crude birth rate (CBR), general fertility rate, age-specific/marital fertility rates, total/marital fertility rates, gross reproduction rate, mean childbearing age, and sex ratio at birth. Mortality rates, a critical facet of the analyses, incorporated metrics like crude death rate (CDR), maternal mortality rate, infant mortality rate, child mortality rate (commonly referred to as “under-five mortality rate”), standard mortality rate (SMR) stratified by age and gender, age-specific mortality rate (ASMR), and life expectancy. Furthermore, the dataset also allows for the computation of migration rates, adding another layer of insightful demographic analysis.

## 4 Method

This section explores a detailed description of the methodologies employed for the migration of the source INDEPTH Core Micro Dataset to the Observational Medical Outcomes Partnership Common Data Model (OMOP CDM). The migration process involves a systematic and comprehensive approach aimed at ensuring the integration and representation of the INDEPTH Core Micro Dataset within the standardised framework of the OMOP CDM.

The intricacies of the migration methodology encompass a series of well-defined steps, including but not limited to data profiling, vocabulary mapping, extraction, transformation, and loading (ETL) programs. Each step is carefully coordinated to maintain the reliability of the data while aligning it with the standardised structure and semantics prescribed by the OMOP CDM. The documentation of these methods provides a transparent and replicable framework, essential for understanding the transformation journey of the data and ensuring the reliability and consistency of subsequent analyses within the OMOP CDM.

### 4.1 INDEPTH core microdata

The key structure of the INDEPTH Core Microdata (11) is described below in Table 1 and Table 2.

**Table 2:** Description of events in the INDEPTH core microdata.

Event	Code	Definition
Birth	BTH	The birth of an individual to a resident female
Enumeration	ENU	Starting event individuals present at the baseline census of the surveillance area.
In-migration	IMG	Migrating into the surveillance area
Out-migration	OMG	Migrating out of the surveillance area
Location exit	EXT	Leaving a residential location to move within the surveillance area
Location entry	ENT	Moving into a residential location after the location exit within the surveillance area
Death	DTH	Death of an individual within the surveillance area
Delivery	DLV	Pregnancy ends after 28 weeks of gestation which may or may not result in birth
Observation end	OBE	An event is inserted when a data set is right censored at an arbitrary date and the individual remains within the surveillance area beyond this date.
Last Observation	OBL	An event indicating the last point in time when the individual was observed within the surveillance area.
Observation	OBS	Recorded characteristics of the individual like educational attainment, employment status, etc. under the surveillance area

The table below describes the categories of events documented in the INDEPTH Core Microdata.

### 4.2 Source data profile

The three source data sets taken from the INDEPTH Data Repository are from Nairobi Urban HDSS from Kenya, Kisesa/Magu HDSS in Tanzania, and Africa Health Research Institute HDSS in South Africa. A brief profile in terms of the population distribution of the datasets that have been extracted from the INDEPTH Data Repository is given in Table 3 and other summaries in Table 4.

**Table 3:** Population distribution in the three datasets.

HDSS site	Male	Female	Other	Total
Kenya—Nairobi	118,487	97,907	0	216,394
Tanzania—Magu(Kisesa)	49,302	56,295	35	105,632
South Africa—AHRI	89,244	106,002	0	195,246
Total	257,033	260,204	35	517,272

**Table 4:** Other summaries from the three datasets.

HDSS site	Events	Live population	First event	Last event
Kenya—Nairobi	741,810	61,486	2003-01-01	2015-12-31
Tanzania—Magu(Kisesa)	203,994	34,832	1994-08-01	2010-12-31
South Africa—AHRI	494,380	109,329	2000-01-01	2017-12-31
Total	1,440,184	205,647		

It is important to highlight that all the INDEPTH Micro Datasets adhere to the same structure and vocabulary list. In this particular use case, we have focused on three specific sites to illustrate the pipeline originating from the INDEPTH Micro Dataset, which is regarded as the designated *exchange protocol* for standard Health and Demographic Surveillance System (HDSS) variables to the Observational Medical Outcomes Partnership Common Data Model (OMOP CDM). Consequently, any dataset adhering to this established *exchange protocol* can seamlessly participate in the standardised pipeline for the efficient migration of data into the OMOP CDM.

**Figure 3:**
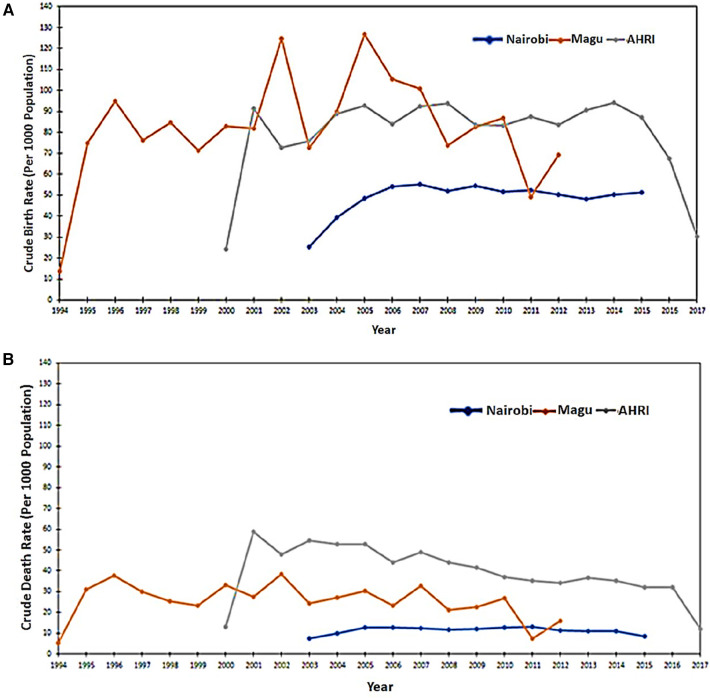
(**A**) Crude birth rates as extracted from the three input datasets. (**B**) Crude Death Rates as extracted from the three input datasets.

Figures 3A,B analyses the data extracted from the INDEPTH Data Repository across three sites: Nairobi, Magu, and AHRI HDSS. Figure 3A shows the Crude Birth Rates and Figure 3B shows the Crude Death Rates.

### 4.3 INSPIRE datahub implementation

The INSPIRE datahub implemented the OMOP CDM using the INDEPTH datasets as a case study. This included data profiling, vocabulary mapping, ETL development, OMOP-based data validation, analysis, and visualization.

**Figure 4:**
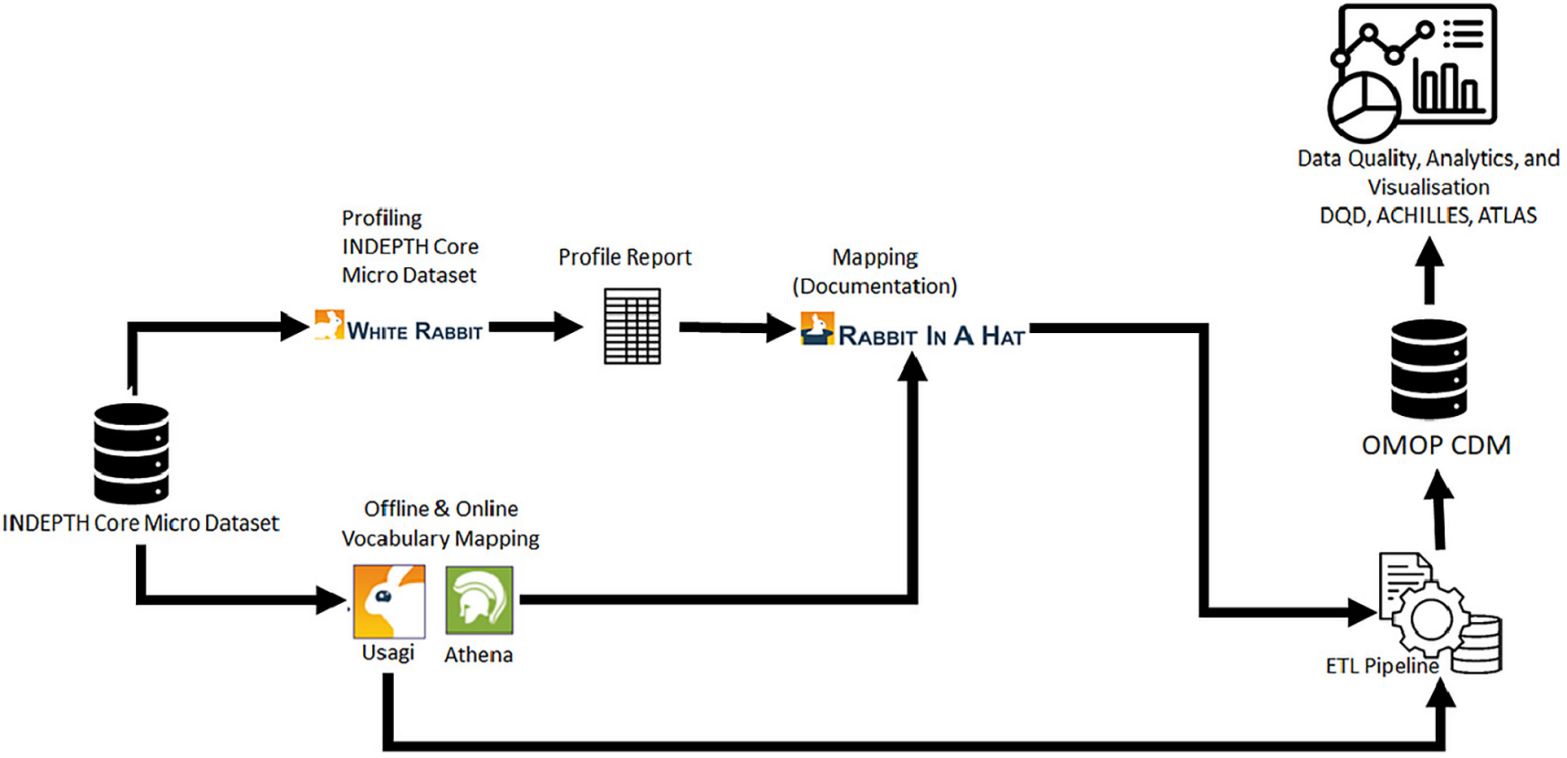
Demonstration of the data flow from INDEPTH data repository to INSPIRE and to OMOP CDM.

Figure 4, depicted below, illustrates the data flow from the INDEPTH Data Repository (28) to the INSPIRE data platform (datahub). Here, the INSPIRE datahub extracts data from the INDEPTH Data Repository, navigating it through various processes to populate an Observational Medical Outcomes Partnership Common Data Model (OMOP CDM) instance. The resultant data is then visualized on the ATLAS dashboard.

### 4.4 Mapping INDEPTH core microdata to OMOP CDM

In this section, we explore the intricate process of mapping source data codes to the standardised vocabularies available on the OHDSI ATHENA repository. We also used the USAGI tool from the OHDSI software stack. USAGI plays a role in streamlining the manual task of code mapping by creating an index of codes obtained from the ATHENA repository. This index serves as a foundation for the creation of code mappings, ensuring alignment with the OHDSI standards.

Following the code mapping process, we transition to elucidating the design details of data mapping. This involves leveraging the dynamic capabilities of WHITERABBIT and RABBIT-IN-A-HAT tools, both integral components of the OHDSI software stack. WHITERABBIT facilitates the exploration and profiling of data, whereas RABBIT-IN-A-HAT enables the systematic creation of data mappings, ensuring a harmonious alignment with the standardised structure of the Observational Medical Outcomes Partnership (OMOP) Common Data Model (CDM). This holistic approach ensures the precision and reliability of the mapping process, laying the groundwork for effective integration and analysis within the OHDSI ecosystem.

Table 5 provides a detail of the source to OMOP standard vocabulary mapping, an output generated through the utilization of the USAGI tool. USAGI, an integral component of the OHDSI software stack, played a role in this process. It systematically helped to create the mappings by employing an indexed foundation of codes derived from the OHDSI ATHENA repository. The result is a clear and structured representation that enhances the understanding and accessibility of data within the OHDSI ecosystem, laying the groundwork for consistent and interoperable analyses.

**Table 5:** Vocabulary mapping from INDEPTH core microdata to OMOP CDM.

Source_ code	Source_ concept_id	Source_vocabulary_id	Source_code_ description	Target_ concept_id	Target_concept_ name	Target_ vocabulary_id	Valid_start_ date	Valid_end_ date	Invalid_ reason
1	0	INDEPTH to OMOP CDM	Male	8507	MALE	Gender	01-01-1970	31-12-2099	
2	0	INDEPTH to OMOP CDM	Female	8532	FEMALE	Gender	01-01-1970	31-12-2099	
3	0	INDEPTH to OMOP CDM	Unknown	8551	UNKNOWN	Gender	01-01-1970	31-Jul-2014	Deprecated
404	0	INDEPTH to OMOP CDM	Kenya	4075204	Kenya	SNOMED (Class = Location)	01-01-1970	31-12-2099	
710	0	INDEPTH to OMOP CDM	South Africa	4073743	South Africa	SNOMED (Class = Location)	01-01-1970	31-12-2099	
834	0	INDEPTH to OMOP CDM	Tanzania	4072112	Tanzania	SNOMED (Class = Location)	01-01-1970	31-12-2099	
BTH	0	INDEPTH to OMOP CDM	Birth	4014291	Birth detail	SNOMED	01-01-1970	31-12-2099	
DLV	0	INDEPTH to OMOP CDM	Delivery	40766614	Outcome of pregnancy	LOINC	01-01-1970	31-12-2099	
DTH	0	INDEPTH to OMOP CDM	Death	4306655	Death	SNOMED	01-01-1970	31-12-2099	
ENT	0	INDEPTH to OMOP CDM	Location entry	4250920	Migration [Qualifier = 4127806 (Internal)]	SNOMED	01-01-1970	31-12-2099	
ENU	0	INDEPTH to OMOP CDM	Enumeration	4251908	Screening Surveillance	SNOMED	01-01-1970	31-12-2099	
EXT	0	INDEPTH to OMOP CDM	Location exit	4250920	Migration [Qualifier = 4127806 (Internal)]	SNOMED	01-01-1970	31-12-2099	
IMG	0	INDEPTH to OMOP CDM	In-migration	4250920	Migration [Qualifier = 4130085 (External)]	SNOMED	01-01-1970	31-12-2099	
OBE	0	INDEPTH to OMOP CDM	Observation end	4129948	End	SNOMED	01-01-1970	31-12-2099	
OBL	0	INDEPTH to OMOP CDM	Last observation	4129948	End	SNOMED	01-01-1970	31-12-2099	
OBS	0	INDEPTH to OMOP CDM	Observation	4129948	End	SNOMED	01-01-1970	31-12-2099	
OMG	0	INDEPTH to OMOP CDM	Out-migration	4250920	Migration [Qualifier = 4130085 (External)]	SNOMED	01-01-1970	31-12-2099	
UNK	0	INDEPTH to OMOP CDM	Unknown	4129922	Unknown	SNOMED	01-01-1970	31-12-2099	

In the source dataset, none of the concepts were defined within the scope of OHDSI's standard concepts. Consequently, the source_concept_id is consistently recorded as 0 for all variable mappings. This adherence to assigning 0 as the source_concept_id is a common practice in OMOP CDM mappings, especially when the concepts originating from the OMOP vocabulary are not explicitly defined within the source dataset(s). This standardised approach ensures clarity and consistency in instances where specific concepts may not be directly mapped from the source, maintaining transparency in the mapping process within the Observational Medical Outcomes Partnership Common Data Model (OMOP CDM).

### 4.5 Extract, transform, and load INDEPTH core microdata to OMOP CDM

The OMOP CDM is a structured and normalized database model designed to facilitate the analysis of real-world healthcare data for observational research, including safety and outcomes studies. In this case study, we demonstrate the mapping and migration of population demographic data into OMOP CDM to highlight the adaptations done to complement this type of data.

The breakdown of each step in the ETL process includes:

Extract: In this phase, data from the three HDSS sites were downloaded from the INDEPTH Data Repository. The downloaded data was in comma-separated file (.csv) format. After comparing the MD5 hash, for content verification, with the original and the download files, data was pushed into PostgreSQL (29) database schema for staging purposes. The comparison of the MD5 values confirmed the content and no data loss or corruption during and post download. A brief data profiling with the statistics displayed on the INDEPTH Data Repository and that of the data in the PostgreSQL schema further helped to document the data validation process and results.

Transform: In this phase, the following tasks were performed:

(1) *Mapping*: Mapping was performed using the OHDSI Rabbit-in-a-Hat tool. Rabbit-in-a-Hat produced an ETL design. In this design the data elements from the source were mapped to the corresponding concepts in the OMOP CDM. This involved standardised codes and terminologies, such as SNOMED and LOINC to ensure consistent representation across the HDSS site data. The remaining phases in this step were implemented using the Pentaho Data Integration (PDI) Community Edition (CE) tool (30).
(2) *Standardisation*: Standardization included date formats, uniform code representations, units of measurement, and other data elements that were converted to a consistent format. This was a crucial step for accurate analysis across HDSS data sources.
(3) *Anonymization*: Study participant privacy is important and thus the data must be de-identified to remove any personally identifiable information. The person identifier in the OMOP CDM was a sequential number, unique to everyone in all the datasets but a mapping with the original identifier key was retained for provenance and
(4) *Structural transformation*: the data structure was reshaped from the source single table (single.csv file for each site) structure to a normalized relational database structure of the target OMOP CDM.

Load: In this final phase, the transformed data was loaded into the OMOP CDM on the PostgreSQL database. The target database consisted of the OMOP CDM version 5.4 (31) clinical and health system tables. The vocabulary tables were loaded separately after downloading the concepts from the ATHENA repository.

**Figure 5:**
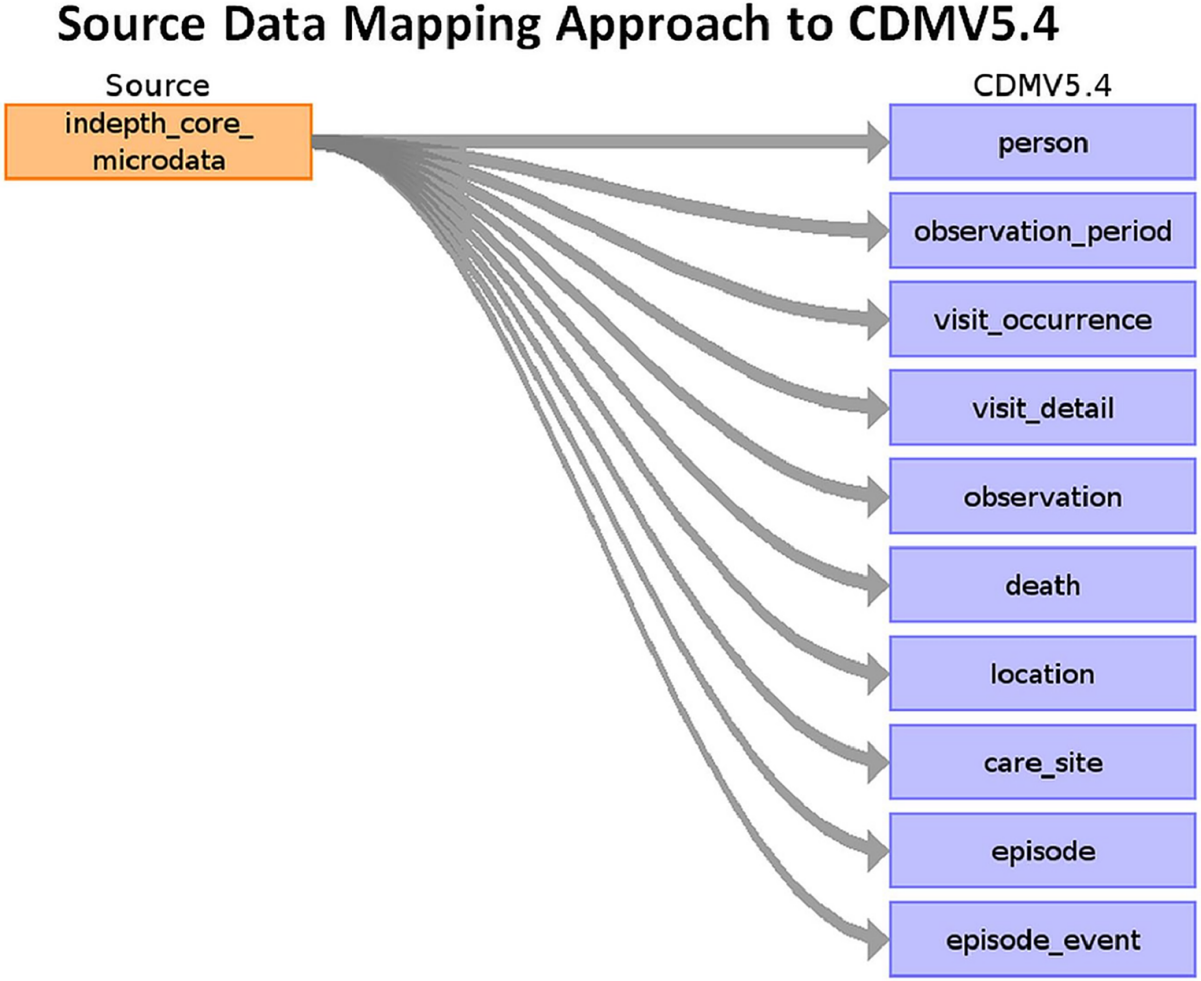
Source data mapping approach to OMOP CDM V5.4.

Figure 5 shows the high-level mapping from INDEPTH Core Microdata to OMOP CDM.

### 4.6 Data migration from INDEPTH to OMOP CDM—the extract, transform, and load implementation

**Figure 6:**
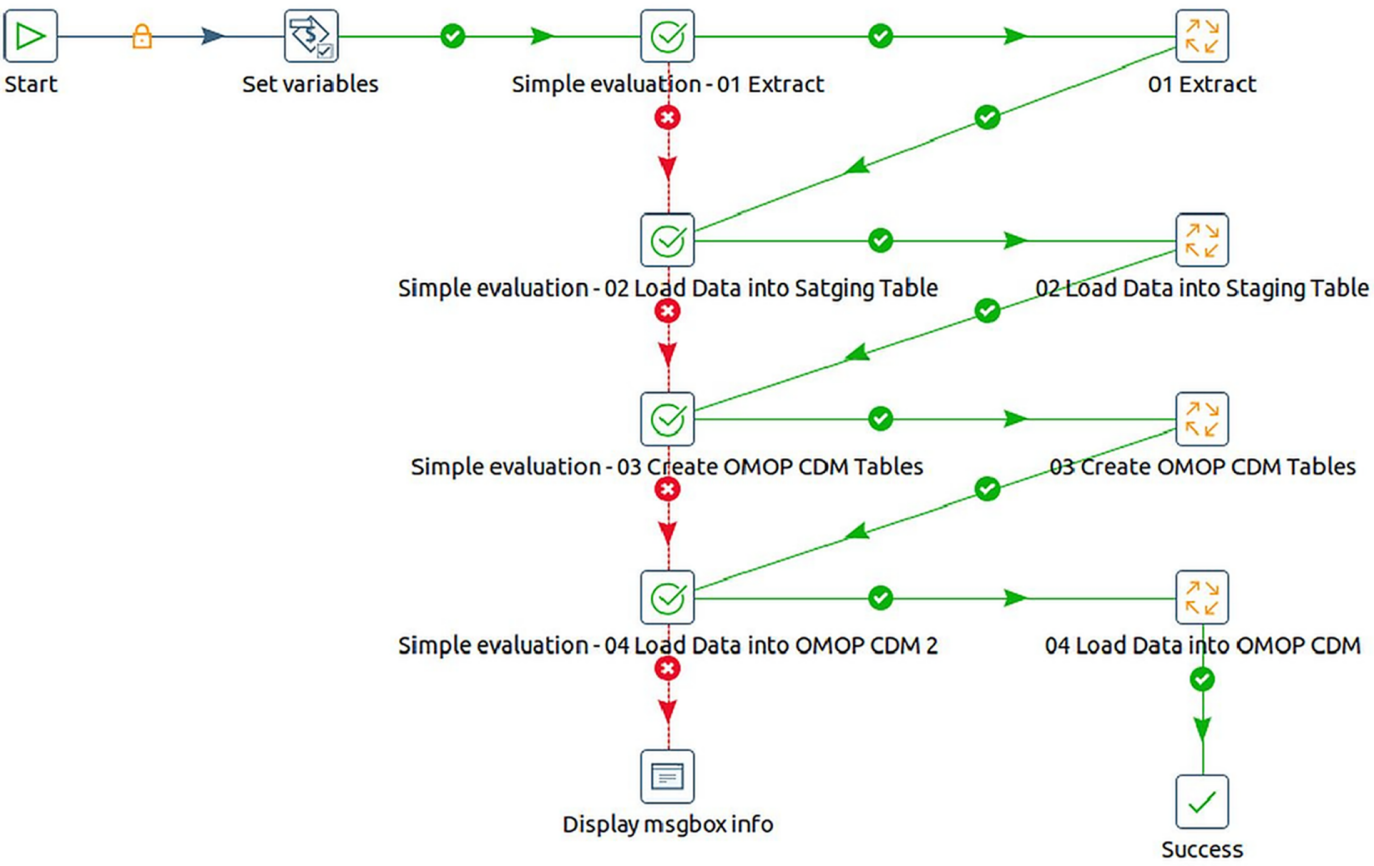
Screenshot of the master job for ETL implementation using pentaho data integration tool.

**Figure 7:**
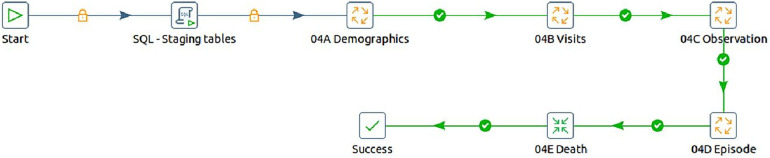
The steps to transform INDEPTH core micro dataset to OMOP CDM.

The ETL process was executed utilizing the Pentaho Data Integration Community Edition tool, with data storage housed within a secure PostgreSQL database environment. The implementation of this process adhered to the following sequential steps:

1. We designed a master Pentaho job to optimize the workflow, enabling seamless coordination of various processes in a sequential chain of jobs and transformations. This approach ensured efficient data migration. Figure 6 shows a screenshot of the master job.
  a. The first job entry does the task of configuring the execution environment for the ETL process. This involves setting variables for database names, schemas, file paths, and other essential parameters.
  b. The Extract process is then responsible for retrieving data from source CSV files, which were previously downloaded from the INDEPTH Data Repository. Subsequently, this data is loaded into corresponding database tables.
  c. In the loading step, data is extracted from the database tables and then merged and loaded into a unified table known as the staging data source. The staging tables are created dynamically before loading the data. This consolidated data serves as the foundation for subsequent processing.
  d. The next entry is focused on creating the OMOP CDM version 5.4 tables within the specified schema for the target database.
  e. The final entry is tasked with transforming the input data from the staging data store into the OMOP CDM format and storing it within the designated target database schema.
2. The final transformation that eventually converts the source data into OMOP CDM performs the following steps, as illustrated in Figure 7:
  a. Here, additional staging tables are created to temporarily store in-process data essential for subsequent steps.
  b. Initial data loading involved demographic details into the OMOP CDM tables, containing individual information and associated data such as location and care site.
  c. In the second step, the process focused on loading visit details, which contained each household encounter. The key conceptual shift from the source data is that source observations are recorded as visits in OMOP CDM. Here, the VISIT_OCCURRENCE and VISIT_DETAIL tables are populated.
  d. During the third step, the OBSERVATION table is populated. This step involved mapping the actual events from the INDEPTH Core Microdata to observations in OMOP CDM, populating both the OBSERVATION and OBSERVATION_PERIOD tables.
  e. In the fourth step, the long-format data from the INDEPTH Core Micro Dataset is transformed into residencies i.e., wide format, signifying the start and end of each residency in a single record. These residencies are then mapped to Episodes in OMOP CDM, resulting in the population of the EPISODE and EPISODE_EVENT tables.
  f. Lastly, the process involved extracting death events from the INDEPTH Core Micro Dataset and populating the DEATH table in OMOP CDM.

### 4.7 Metadata

Metadata serves as an important layer of information that explains the characteristics, context, and origins of data, resources, and information sources. Its main function is to empower users by facilitating a comprehensive understanding of the data, enabling successful utilisation. Positioned as a vital component in data management, discovery, and interpretation, metadata assumes a key role in adhering to the FAIR (Findable, Accessible, Interoperable, Reusable) principles, essential for fostering data sharing and open science.

The benefits of metadata include but are not limited to: (1) expediting data discovery, (2) enhancing data interoperability, (3) facilitating data quality assessment, and (4) enabling proper data citation. In principle, metadata acts as a key enabler, ensuring that data is not only accessible but also comprehensible and usable in diverse contexts.

For a detailed insight into the INSPIRE Data Hub's metadata flows, readers may refer to the comprehensive documentation provided in the “INSPIRE Data Hub: Technical Results of Phase 1” (20). This document explains the workings of the metadata flows within the INSPIRE Data Hub, shedding light on the technical aspects and outcomes of the initial phase.

## 5 Result

The OHDSI Data Quality Dashboard (DQD) serves as a specialized tool for the assessment and continuous monitoring of data quality within the Observational Medical Outcomes Partnership Common Data Model (OMOP CDM). This comprehensive dashboard functions as a strategic overview, empowering analysts to pinpoint and address potential issues residing in the CDM datasets. Leveraging an array of sophisticated data quality checks, the dashboard offers valuable insights into crucial dimensions, including completeness, accuracy, and consistency of the data.

The data quality checks within the OHDSI framework are systematically categorized into three main types: table checks, field checks, and concept-level checks, each addressing distinct aspects of data integrity. Table-level checks operate at a high level, evaluating entire tables without investigating into individual fields. Such checks ensure the presence of required tables or verify that individuals in the PERSON table have corresponding records in event tables. Field-level checks, most check types, focus on specific fields within a table. These encompass assessments of primary key relationships and scrutiny of whether concepts in a field adhere to specified domains. Concept-level checks zoom in on individual concepts, scrutinizing aspects such as the presence of gender-specific concepts in individuals of the wrong gender and the validity of values for measurement-unit pairs (22).

Upon completion of the check process, the R package generates a JSON object which can be viewed using a web browser (22). This multi-tiered approach ensures a comprehensive evaluation of data quality.

One of the key strengths of the dashboard lies in its ability to facilitate improvement efforts by providing a transparent mechanism for tracking and addressing identified issues. This, in turn, ensures that the information housed within the OHDSI data stack maintains a high standard of reliability, making it well-suited for a diverse array of analytical endeavours. Thus, the OHDSI Data Quality Dashboard emerges as a pivotal asset in fortifying the integrity of data within the OMOP CDM, fostering confidence in the data's suitability for robust analytics.

**Figure 8:**
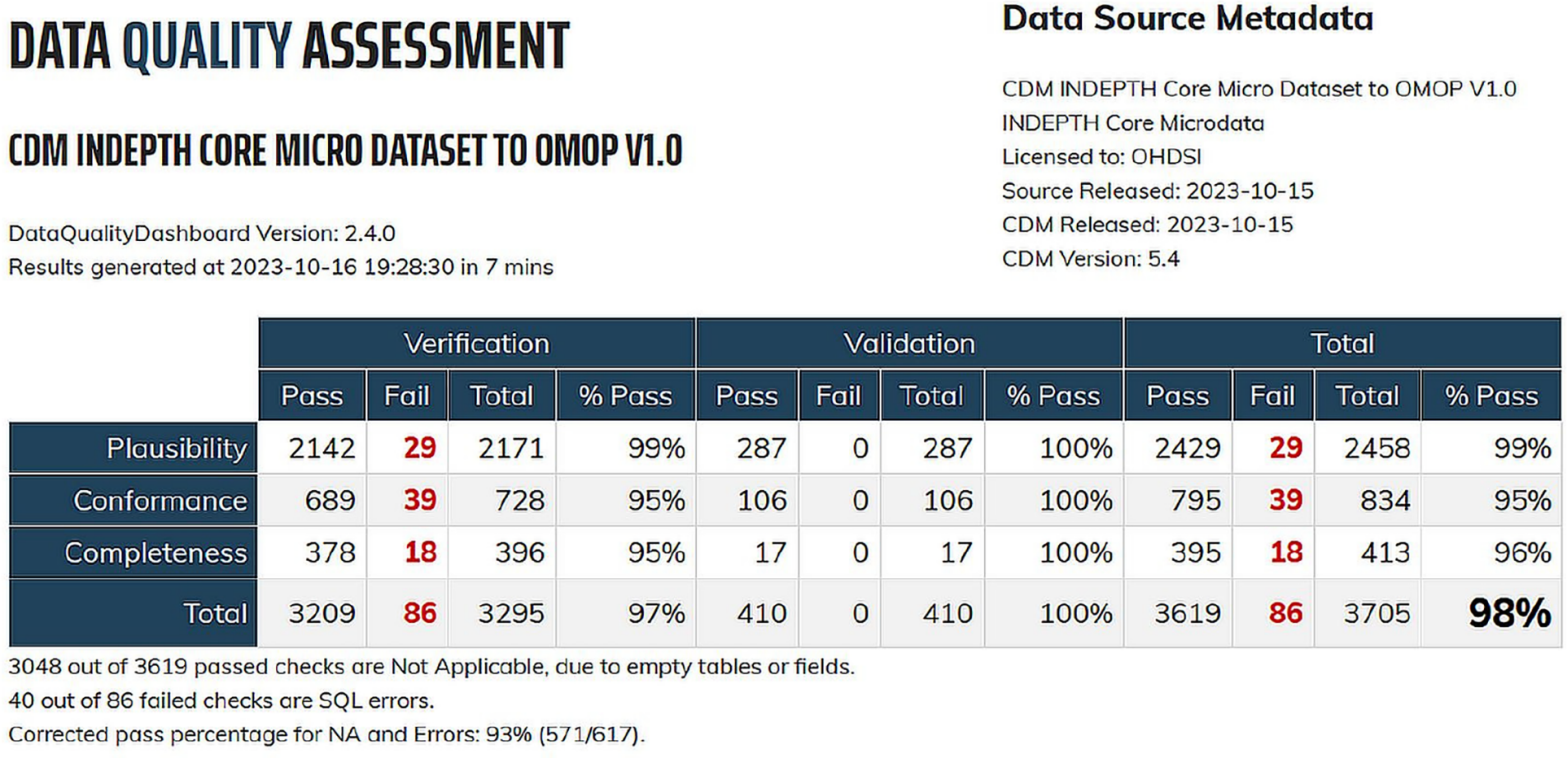
Data quality dashboard for OMOP CDM migration from INDEPTH core micro dataset.

Figure 8 shows the post-migration data quality after transitioning to the OMOP CDM format from the three datasets sourced from the INDEPTH Data Repository.

OHDSI ATLAS is a tool that provides a no code/low code platform for the exploration, analysis, and visualisation of OMOP CDM data. It facilitates the creation of cohort definitions, the exploration of the CDM, and the generation of various statistics and visualisations. It also populates the OMOP CDM results schema. The CDM data post migration from the INDEPTH Core Micro Dataset has been processed using ATLAS and the ACHILLES R scripts it invokes. Together they populated result tables of ATLAS to visually display the results.

**Figure 9:**
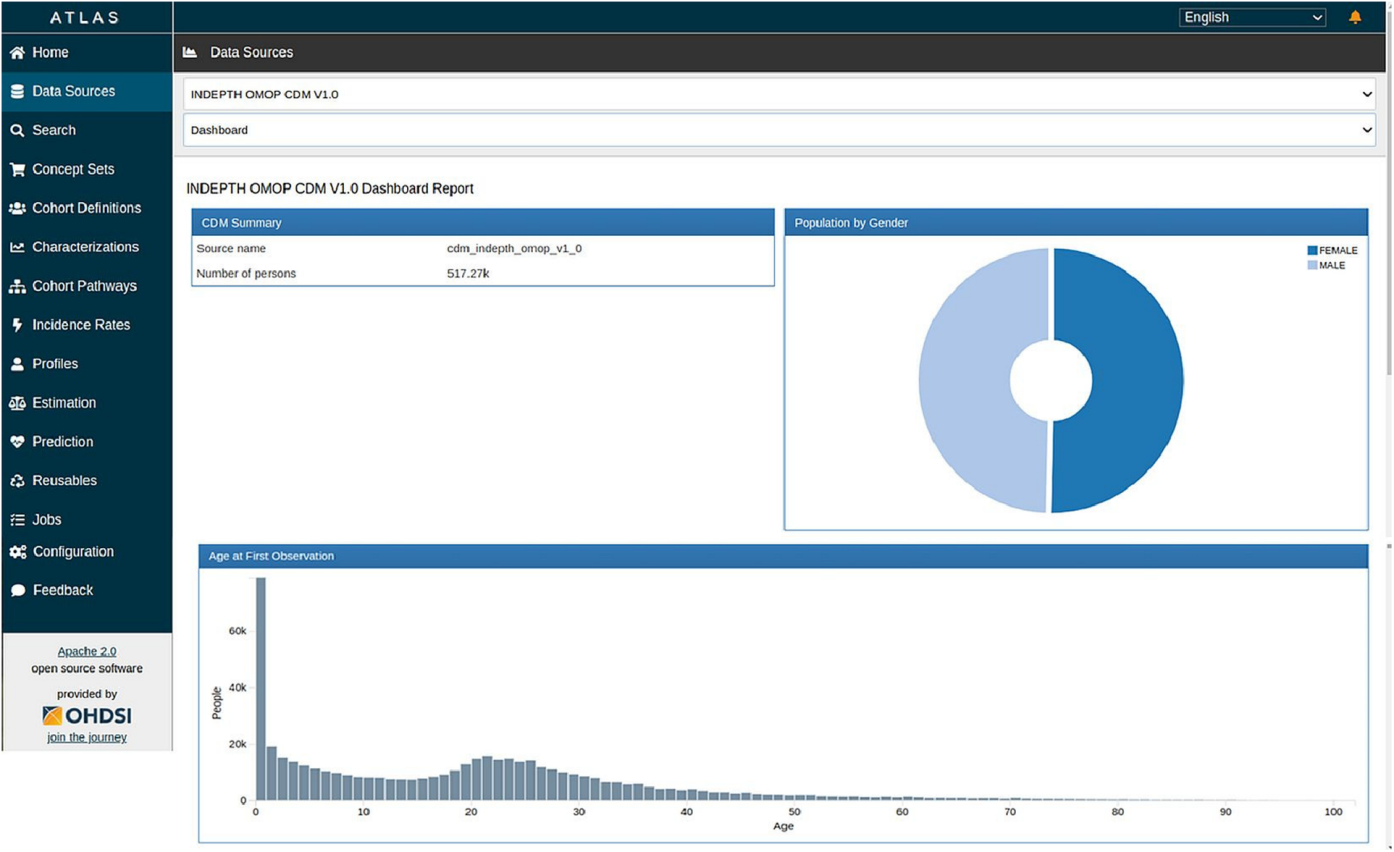
ATLAS dashboard.

**Figure 10:**
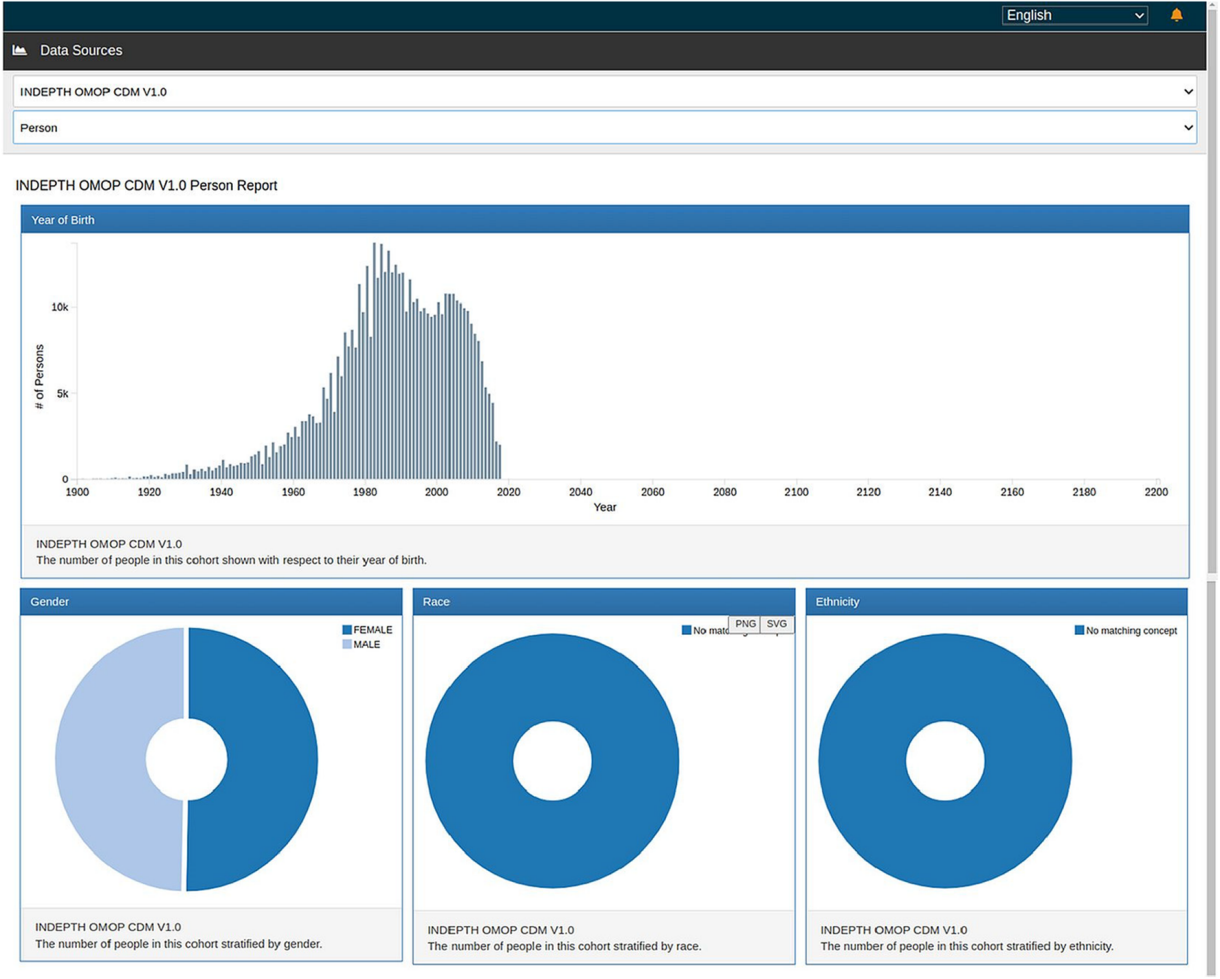
ATLAS person report.

Figures 9, 10 show the ATLAS data dashboard and individual-level PERSON data, respectively.

**Figure 11:**
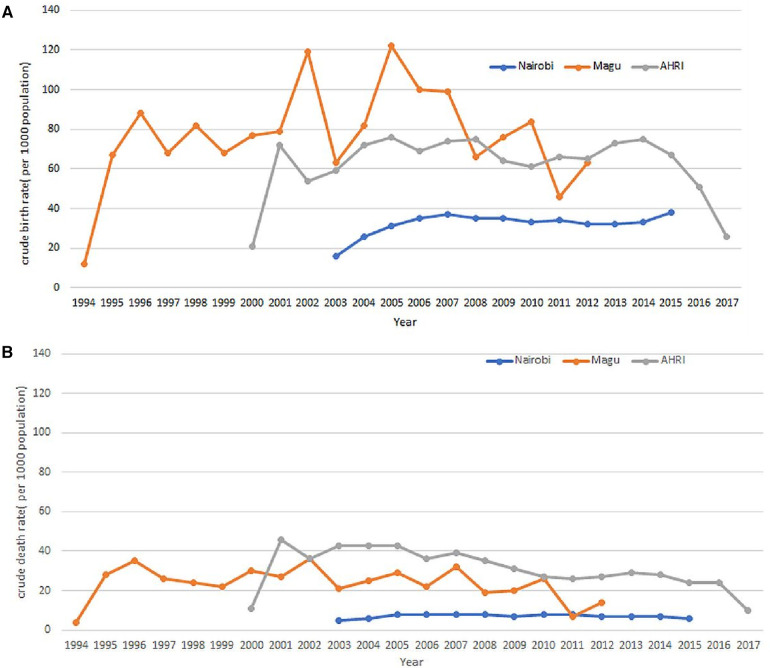
(**A**) Crude birth rates as extracted from the OMOP CDM. (**B**) Crude Death Rates as extracted from the OMOP CDM.

After completing the data migration, an analysis was conducted to compute the crude birth and death rates based on the OMOP CDM dataset. The results of this analysis demonstrated that the rates remained consistent both before and after the data migration. Figures 11A,B visually represent the Crude Birth Rate (CBR) and Crude Death Rate (CDR) following the migration of data into OMOP CDM from the INDEPTH Core Micro Dataset. These figures provide clear evidence that the ETL process was successful, ensuring that no or minimal data loss occurred during the migration.

## 6 Conclusion

In conclusion, this research has explored the processes of data mapping and migration, focusing on the transformation from the INDEPTH Core Micro Dataset to the Observational Medical Outcomes Partnership Common Data Model (OMOP CDM). The systematic approach undertaken in this study highlights the importance of standardising heterogeneous data sources, enabling interoperability, and fostering harmonization within the realm of population health research.

This paper highlights the methodology for setting up an ecosystem, for data harmonising into the OMOP CDM from INDEPTH Core Micro Dataset and does not explore the complex analysis of the datasets we are undertaking post-migration.

Through data profiling, vocabulary mapping, and the execution of extraction, transformation, and loading (ETL) programs, we have successfully navigated the challenges associated with integrating data from an exchange format into the OMOP CDM. The use of tools such as USAGI, WHITERABBIT, and RABBIT-IN-A-HAT from the OHDSI open-source software stack has played a pivotal role in achieving accurate and meaningful mappings, ensuring the fidelity of data representation. It also demonstrated the use of the Pentaho Data Integration tool for implementing the ETL pipeline.

The integration of the OHDSI Data Quality Dashboard into our methodology further fortifies the reliability of the transformed data. By facilitating continuous monitoring and assessment, this tool empowers researchers to uphold the quality, completeness, and accuracy of the migrated data, laying a robust foundation for subsequent analytics and research endeavours.

This research not only contributes to the advancement of data harmonisation practices but also aligns with the broader global initiatives advocating for the adoption of standardised data models. The insights gained from this study have implications not only for the INSPIRE Network but also for the wider research community engaged in longitudinal population studies. The ability to seamlessly migrate data from a diverse range of sources into the OMOP CDM opens avenues for collaborative research, comparative analyses, and evidence generation on a broader scale.

A notable insight from this endeavour is the successful exploration of integrating longitudinal population data into the Observational Medical Outcomes Partnership Common Data Model (OMOP CDM), despite its primary design for clinical data.

The OMOP CDM v5.4 encompasses 15 tables within the Clinical Data Tables. In our study, we populated 6 tables due to the limited availability of data in the source. From the Health System Data Tables, we successfully populated 2 out of the 3 tables, and from the Standardised Derived Elements, we populated 2 out of 5 tables. The omission of certain tables does not compromise the effectiveness of the migration process or the analytical capabilities, as the inclusion of these tables is contingent upon the availability of specific data types. Overall, the migration has proven successful, and standard analyses using the OHDSI tools are both underway and have been already been achieved. The result of the migration is shown in the output screenshots of the Data Quality Dashboard and the ATLAS.

## 7 Discussion

The concept of harmonised data across a network of data providers started with patient data in High-Income Countries (HIC) (32). The INSPIRE datahub is extending that concept to new sources of data, including population health data, building on approaches by INDEPTH (11) and the ALPHA network (4). The established approach is through standardised vocabularies that describe the provenance and metadata. This allows ETL programs to on-ramp the data into a common data model for use with other similar data. In using the larger, more diverse data source low- and middle-income countries (LMIC), there is greater confidence in the results and the applicability to a wider population. The use case shows how demographic data from the iSHARE repository can be made available, so we can use data from 25 HDSS across Africa, providing an opportunity for comparative analysis across African countries. The key to achieving this is firstly the maintenance of the HDSS data collection, and secondly the training of HDSS data managers to undertake the ETL into HDSS instances of the OMOP CDM.

The approach by INSPIRE has been to populate a common data model that is generic, scalable, and displays the rich provenance of metadata from study data. We have used the OMOP common data model as the standard and have adapted it to use with population data. There are still areas where further adaptation is needed. We have shown the whole data pipeline, including visualization and profiling of data, can be achieved, using the case study data from three HDSS. In developing this model, we have developed training modules (https://inspiredata.network/courses) for data managers, to enable them to understand the new concepts in the OMOP CDM. We have started with a basic use case data set, but the principles can be used on other data, and an ETL is needed with each new dataset.

The ALPHA data specification (https://alpha.lshtm.ac.uk/data-2/) and the INDEPTH Core Microdata (https://academic.oup.com/view-large/81282722) are focused on specific structures that are more suitable for analysis of HIV and population studies respectively. They do not cater to a generic structure for all types of studies and do not offer any pre-built tools to prepare and analyse data. In comparison, OMOP CDM and the connected OHDSI community provide open-source tools to prepare and analyse data using a rich set of vocabularies for standardizing population health encounters and their observations from diverse sources.

For capacity building within its collaborating sites and other interested groups, the INSPIRE network has developed training modules (https://www.inspiredata.network/courses) focused on data harmonization. These modules cater to all the aspects of data harmonization including ethics, discovery, anonymization, specifications, exchange protocols, OMOP CDM, migration (ETL), validation, governance, sharing, analysis, and many more. These modules are expected to raise awareness for data harmonization and build the necessary skills within the African sites.

The initial work focused on transforming the ALPHA data specification to OMOP CDM (33). This covered the transformation of data related to HIV status in a population. This effort was taken up as a proof of concept that OMOP CDM can be used for population-based studies although OMOP CDM was primarily created for clinical datasets. The use case taken up in this paper demonstrated the adaptation of OMOP CDM for demographic and health surveillance system data, which augmented the understanding of modifying the initial purpose of OMOP CDM into a new area of study. Putting HDSS data into OMOP increased the scope of demographic data to be aligned and researched alongside diseases and clinical data to give better insight into public health questions.

The OHDSI open-source software stack (https://ohdsi.org/software-tools/) gives its users a variety of tools to prepare, analyse and visualise data. It provides skeletal SQL and R codes to implement the data migration (ETL) design. However, the skeletal codes are not sufficient to implement the data migration and more so in complex situations, the codes need to be modified, which requires specialized skills. In INSPIRE, for this case study, we have used the Pentaho Data Integration (PDI) Community Edition (CE) tool to implement the data migration process taking inputs from the designs done using the OHDSI WhiteRabbit tools. Pentaho Data Integration (PDI) Community Edition (CE) (30) is an open-source version from Hitachi Vantara, which is a graphical tool designed specifically for data migration (ETL) that can handle complex transformation logics and large datasets through its inbuilt features and multi-threaded architecture.

Since OMOP CDM was initially designed and developed to harmonise clinical data, the data standards, including table structures, variables, and codes were more specific to the clinical environment. When doing the data mappings for ALPHA data specifications, INDEPTH Core Microdata, and other INSPIRE studies, it was realised at times that the vocabulary do not suffice to population study data completely. Some mappings were done on a very generic basis as specific vocabularies were not found in the ATHENA vocabulary repository. Therefore, it is recommended to add additional vocabularies to serve a diverse type of datasets including population studies and indigenous terminologies specific to the African context. Additional vocabularies are part of the purview of the OHDSI Africa Chapter Working Group.

The INSPIRE datahub will help to understand and make progress in setting up data hubs within Africa with low resource settings to achieve data harmonization for access to global research opportunities and data sharing. A scalable architecture, as has been proposed and demonstrated by INSPIRE PaaS will enable sites to replicate or use a centralized host to process and analyse data. The iSHARE (28) and ALPHA have demonstrated the use of Centre-in-a-Box (CiB) (5), an all-encompassing box for research data management. The CiB ensured site autonomy concerning data by enabling all data preparation work to be done within the site premises and then sharing the anonymized datasets in a common standard on the INDEPTH Data Repository (https://indepth-ishare.org/). This was the beginning of a federated workflow within African HDSS sites. INSPIRE datahub is working towards achieving a federated data network on a public/private cloud and an on-premises combination of servers. The other benefit that is accounted for in this data hub is the process of data FAIRfication. The work to FAIRify OMOP CDM is underway.

The case study presented herein underscores the compelling effectiveness of the OHDSI framework in seamlessly harmonizing heterogeneous data sources. Through meticulous analysis, it becomes evident that the OHDSI framework adeptly bridges disparities inherent in diverse data origins, culminating in a unified and standardised repository. This unification greatly enhances the feasibility of data comparison and aggregation, fostering an environment conducive to robust healthcare analytics and research.

Moreover, the study illustrates the remarkable interoperability achieved by the OHDSI framework in facilitating the integration of transformed data with other tools and databases adhering to the OHDSI compatibility standards. The findings underscore the pivotal role of this interoperability in creating a synergistic ecosystem, allowing for the seamless exchange of insights and analyses across various platforms. This not only expedites research processes but also augments the reliability and reproducibility of results, a critical facet of any data-driven endeavor.

This pan-African data hub will benefit researchers to work on multi-site data and produce results affecting the entire region rather than just surveillance area-specific locations. This will ensure decisions related to public health cover larger areas and regions improving lives therein.

As part of the platform's ongoing development, we are currently working on the use of OHDSI Docker images at HDSS sites. This effort aims to introduce read-only templates that provide step-by-step instructions for creating containers. By doing so, we are streamlining the packaging of tools with all the essential components required to replicate and run seamlessly in other setups.

The limitations of this data hub, as it stands currently, lie in the scope of expansion into other sites. The agreements for using real data from sensitive domains like COVID-19 and HIV remain a challenge due to the concerns that the data owners carry. Unless this datahub, an integrated suite of services, is expanded into new sites and domains, the real test remains elusive.

The future work planned for this integrated suite of services are in deploying OHDSI instances at the collaborating sites using Docker and/or other technologies, adding population study-related and indigenous terminologies into the OMOP standard vocabularies, FAIRification of OMOP CDM, and in the process improving on the metadata generation and documentation, conducting additional analyses at each site using the OHDSI data analysis toolset and hosting multi-domain data results on the hub.

## 8 Future

As we move forward, the lessons learned from this research can guide future endeavours in enhancing data interoperability, supporting reproducibility, and promoting the overarching principles of the FAIR (Findable, Accessible, Interoperable, Reusable) data framework. Ultimately, this study contributes to the evolving landscape of population health research, where standardised data models play a pivotal role in unlocking the potential of diverse datasets for the betterment of global public health.

The INSPIRE data hub currently houses data sourced from the ALPHA data specification, INDEPTH Core Micro Dataset, and synthetic WHO IDSR data about COVID-19 in the African region. Ongoing efforts are directed towards incorporating climate and mental health data into the platform. These additional datasets will undergo processing and integration into the OMOP CDM for analysis and visualization.

Moreover, engagement for enhancing the federated platform's capabilities to accommodate HDSS datasets within the INSPIRE network is in progress. This expansion will enable use cases like sharing of data for conducting analyses and exploring the impact of climate change on community health.

## Data Availability

The original contributions presented in the study are included in the article/Supplementary Material, further inquiries can be directed to the corresponding authors.
